# The Relationship Between Body Mass Index and In-hospital Survival in Patients Admitted With Acute Heart Failure

**DOI:** 10.3389/fcvm.2022.855525

**Published:** 2022-04-28

**Authors:** Gabby Elbaz-Greener, Guy Rozen, Shemy Carasso, Merav Yarkoni, Harindra C. Wijeysundera, Ronny Alcalai, Israel Gotsman, Eldad Rahamim, David Planer, Offer Amir

**Affiliations:** ^1^Department of Cardiology, Hadassah Medical Center, Jerusalem, Israel; ^2^Faculty of Medicine, Hebrew University of Jerusalem, Jerusalem, Israel; ^3^Cardiology Division, Hillel Yaffe Medical Center, Hadera, Israel; ^4^The Ruth and Bruce Rappaport Faculty of Medicine, Technion, Haifa, Israel; ^5^Cardiology Division, Harvard Medical School, Massachusetts General Hospital, Boston, MA, United States; ^6^Division of Cardiovascular Medicine, Baruch Padeh Medical Center, Poriya, Israel; ^7^The Azrieli Faculty of Medicine in the Galilee, Bar-Ilan University, Safed, Israel; ^8^Division of Cardiology, Schulich Heart Centre, Sunnybrook Health Sciences Centre, University of Toronto, Toronto, ON, Canada

**Keywords:** body mass index, BMI, acute heart failure, AHF, outcome

## Abstract

**Background:**

The association between Body Mass Index (BMI) and clinical outcomes following acute heart failure (AHF) hospitalization is debated in the literature. Our objective was to study the real-world relationship between BMI and in-hospital mortality in patients who were admitted with AHF.

**Methods:**

In this retrospective, multi-center study, we utilized the National Inpatient Sample (NIS) database to identify a sampled cohort of patients who were hospitalized with AHF between October 2015 and December 2016. Outcomes of interest included in-hospital mortality and length of stay (LOS). Patients were divided into 6 BMI (kg/m^2^) subgroups according to the World Health Organization (WHO) classification: (1) underweight ≤ 19, (2) normal weight 20–25, (3) overweight 26–30, (4) obese I 31–35, (5) obese II 36–39, and (6) extremely obese ≥40. A multivariable logistic regression model was used to identify predictors of in-hospital mortality and to identify predictors of LOS.

**Results:**

A weighted total of 219,950 hospitalizations for AHF across the US were analyzed. The mean age was 66.3 ± 31.5 years and most patients (51.8%) were male. The crude data showed a non-linear complex relationship between BMI and AHF population outcomes. Patients with elevated BMI exhibited significantly lower in-hospital mortality compared to the underweight and normal weight study participants (5.5, 5,5, 2,8, 1.6, 1.4, 1.6% in groups by BMI ≤ 19, 20–25, 26–30, 31–35, 36–39, and, ≥40 respectively, *p* < 0.001) and shorter LOS. In the multivariable regression model, BMI subgroups of ≤ 25kg/m^2^ were found to be independent predictors of in-hospital mortality. Age and several comorbidities, and also the Deyo Comorbidity Index, were found to be independent predictors of increased mortality in the study population.

**Conclusion:**

A reverse J-shaped relationship between BMI and mortality was documented in patients hospitalized for AHF in the recent years confirming the “obesity paradox” in the real-world setting.

## Introduction

Body mass index (BMI) is widely used for routine characterization of weight status in epidemiological and clinical research. However, it has limitations, for instance, not distinguishing fat from muscle mass (1–3).

According to the World Health Organization (WHO) classification, patients are considered (1) underweight when they have a BMI ≤ 18.5 kg/m^2^, (2) normal weight with BMI levels 18.5–24.9 kg/m^2^, (3) overweight BMI 25–29.9 kg/m^2^, (4) class I obesity BMI 30–34.9 kg/m^2^, (5) class II obesity BMI 35–39.9 kg/m^2^, and (6) extremely obese BMI ≥40 kg/m (2, 4, 5).

Recent evidence suggests that obesity is associated with better outcomes in patients with heart failure (HF) (6). Multiple retrospective studies demonstrated conflict results. Some have shown a U-shape curve between BMI and long-term mortality in patients with acute HF (AHF), paradoxically protecting the obese population (7–17). In some analyses, the U-shape relationship would no longer be apparent after adjusting for confounding prognostic factors of mortality (7, 9). In other studies, the U-shaped BMI remained an independent predictor of mortality even after adjusting for other risk variables in the obese (13, 14) and underweight groups (15).

As the impact of obesity on AHF outcomes is still in debate. There is importance to understand the relationship between obesity and HF to find the optimal treatment goals for our patients. This is especially interesting in the light of the connection between obesity and HF with preserved ejection fraction, an important condition with many question marks. The current study aimed to describe the BMI distribution and its potential relation with in-hospital mortality outcome in six different BMI subgroups of patients with AHF in the large cohort of US database from the National Inpatient Sample (NIS) registry.

## Methods

### Data Source

Pulled data from the NIS, the Healthcare Cost and Utilization Project (HCUP), and Agency for Healthcare Research and Quality (AHRQ) (4, 5) were used for the study. This study was exempt from institutional review by the Human Research Committee due to the NIS database, which only includes de-identified information.

The NIS is the largest collection of data on all-payer hospitalizations in the United States (US) and represents an approximately 20% stratified sample of all inpatient discharges from US hospitals (18). This includes records at the hospital level, such as hospital region, teaching status, bed size, and cost of hospitalization, and other data at the patient level, including demographic characteristics, primary and secondary diagnoses and procedures, comorbidities, and length of stay (LOS). LOS analysis included all cohort populations, i.e., discharged and those who died in the hospital. National estimates can be calculated using the patient-level and hospital-level sampling weights that are provided by the HCUP (4, 5).

For the purpose of this study, we acquired data for the years 2015 and 2016. During the study period, we used the International Classification of Diseases, 10th Revision, Clinical Modification (ICD-10-CM) from the last quarter of 2015 and thereafter for reporting diagnoses and procedures in the NIS database. For each index hospitalization, the database presents a primary discharge diagnosis, a maximum of 14 or 24 additional diagnoses, and a maximum of 15 procedures. For our study, we limited our cohort to the time-period which used ICD-10 codes to convert the data because the ICD-10 coding system includes individual codes for all BMI values and ranges.

### Study Population and Variables

We included patients 18 years or older with a principal diagnosis of AHF hospitalization based on ICD-10-CM codes starting with I50.21, I50.23, I50.31, I50.33, I50.41, I50.43, and I50.811 who have one of the Z68.x codes as I10-Dx1 to I10-Dx30. These codes represent the six subgroups in our study, namely, Z68, Z68.20–25, Z68.26–30, Z68.31–35, Z68.36–39, and Z68.4 (BMI ≤ 19 underweight group; BMI 20–25 normal weight group; BMI 26–30 overweight group; BMI 31–35 obese I group; BMI 36–39 obese II group; and BMI equal or ≥40 extremely obese group, respectively).

Patient demographics were collected from the database as follows: age, sex, and race. Prior comorbidities were identified by measures from the AHRQ. For the purpose of calculating the Deyo-Charlson Comorbidity Index (Deyo-CCI), additional comorbidities were identified from the database using ICD-10-CM codes. Deyo-CCI is a modification of the Charlson Comorbidity Index, containing 17 comorbid conditions of differential weights, with a total score ranging from 0 to 33 (detailed information on Deyo-CCI is provided in Appendix Table 1). Higher Deyo-CCI scores indicate a greater burden of comorbid diseases and are associated with mortality 1-year after admission (19). The index has been used extensively in studies from administrative databases, with proven validity in predicting short- and long-term outcomes (20, 21).

Our primary outcome in this study was in-hospital mortality. The secondary outcomes included length of stay in the hospital.

### Statistical Analysis

The chi-square (χ^2^) and Wilcoxon Rank Sum tests were used to compare categorical variables and continuous variables, respectively. The NIS provides discharge sample weights that are calculated within each sampling level as the ratio of discharges in the universe to discharges in the sample (22). We generated a weighted logistic regression model to identify independent predictors of in-hospital mortality. Our primary covariate of interest was BMI category, with normal weight, with the BMI 20–25 category used as the reference. Candidate variables included patient-level characteristics, Deyo-CCI, and hospital-level factors. We included all candidate variables that were associated with our primary outcome in our final multivariable regression model.

For all analyses, we used SAS^®^ software version 9.4 (SAS Institute Inc., Cary, NC.), and a *p*-value < 0.05 was considered statistically significant.

## Results

### Study Cohort

A total of 43,990 AHF hospitalizations across the US during 2015–2016 were included in the analysis. After applying the weighting method, these represented an estimated total of 219,950 AHF hospitalizations in patients with complete BMI data. Most patients (51.8%) were male, and the cohort's mean age was 66.3 ± 31.5 years.

### AHF Patient Characteristics and Comorbid Presentation by BMI Group

Frequency distribution of baseline and clinical characteristics are presented in Table 1 according to the six BMI-divided subgroups. The median BMI in the study was 39 (IQR: 32–43), with 79.9% of the patients with BMI above the normal (>25kg/m^2^).

**Table 1:** Frequency distribution of baseline characteristics by body mass index (BMI) group in patients with acute heart failure.

**BMI groups** **Kg/m^**2**^**	**≤19** **Under-weight**	**20–25** **Normal-weight**	**26–30** **Over-weight**	**31–35** **Obese I group**	**36–39** **Obese II group**	**≥40** **Extremely obese**	**Total**	***P*-value**
Unweighted^a^	2,784 (6.3)	2,529 (5.8)	3,545 (8.1)	7,159 (16.3)	6,248 (14.2)	21,725 (49.4)	43,990	
Weighted^b^	13,920	12,645	17,725	35,795	31,240	108,625	219,950	
**Age group, %, years**								<0.001
18–44	2.6	2.2	3.6	4.6	6.6	10.1	7.3	
45–59	7.5	8.5	14.0	18.8	23.2	29.9	23.2	
60–74	23.7	24.6	33.7	40.0	41.6	42.7	39.1	
>75	66.2	64.7	48.7	36.6	28.6	17.3	30.4	
**Gender, %**								<0.001
Male	61.8	48.7	44.3	44.3	46.8	56.1	51.8	
Female	38.2	51.2	55.6	55.7	53.2	43.9	48.1	
**Race, %**								<0.001
White	69.3	67.3	64.1	63.1	62.9	60.9	62.7	
Non-white	25.6	27.4	31.1	31.8	32.1	34.0	32.2	
**Comorbidity, %**								
Hypertension	38.3	36.4	38.5	41.6	43.9	43.5	42.1	<0.001
Diabetes mellitus	17.2	25.7	44.1	49.3	51.8	53.7	48.0	<0.001
Chronic renal disease	39.5	46.7	50.9	48.9	46.5	44.8	46.0	<0.001
Chronic obstructive pulmonary disease	46.6	37.3	41.3	45.0	47.6	52.7	48.6	<0.001
Peripheral vascular disease	23.5	25.4	25.8	24.1	21.3	15.8	19.8	<0.001
Atrial Fibrillation/Flutter	47.4	49.0	45.6	44.6	43.1	40.7	43.0	<0.001
Prior MI	14.0	16.3	18.4	17.8	14.5	11.3	13.8	<0.001
**Deyo-CCI, %**								<0.001
1	6.6	6.7	4.3	4.3	4.6	5.1	5.0	
2 or higher	93.4	93.3	95.7	95.7	95.4	94.9	95.0	
**Outcome**								
Mortality	5.5	5.5	2.8	1.6	1.4	1.6	2.2	<0.001
Length of Stay	6.46 ± 0.15	6.7 ± 0.15	5.9 ± 0.08	5.4 ± 0.08	5.4 ± 0.07	6.0 ± 0.05	5.9 ± 0.04	<0.001

*BMI, Body Mass Index (Kg/m^2^); Deyo-CCI, Deyo-Charlson Comorbidity Index; MI, Myocardial Infraction*.

a *Represents the number of observations in the NIS database*.

b *Represents total national estimates after applying sampling weights*.

Of the AHF hospitalization, the majority of the patients were obese type I (16.3%), type II (14.2), and with morbid obesity (49.4%) (Table 1).

Female predominance, higher rate of patients with Deyo-CCI score above 2, diabetes mellitus 2, hypertension, and hyperlipidemia were found in patients with AHF with BMI higher than 25 kg/m^2^. The lean patients with BMI ≤ 19 kg/m^2^ were predominantly male and were the least diabetic.

The overall in-hospital mortality rate during the study period was documented at 2.2%.

**Figure 1:**
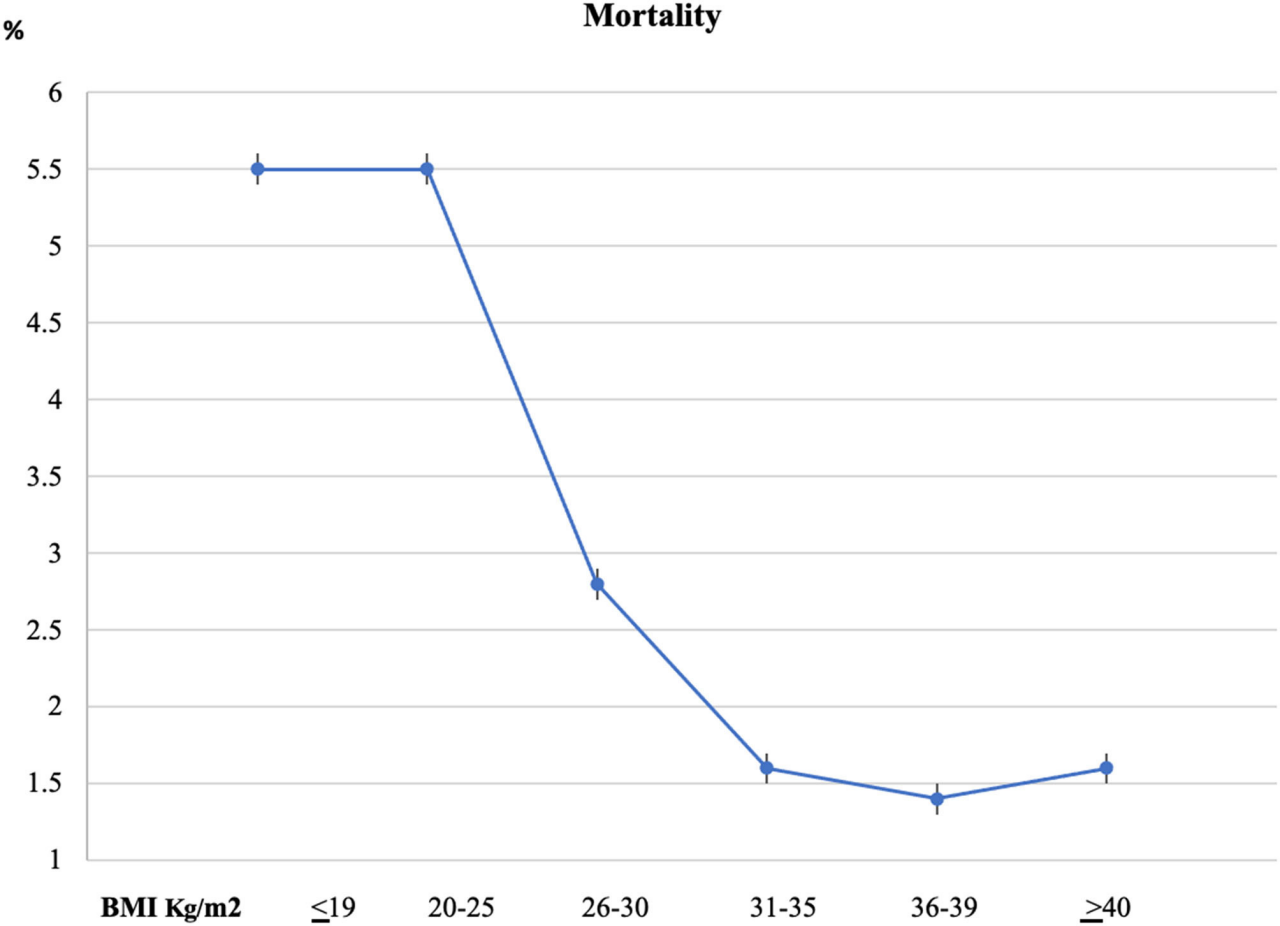
Mortality rate *via* body mass index (BMI) groups.

The highest crude mortality rate was observed in the underweight and normal weight subgroup 5.5% in each group, with decreased mortality rate in patients with BMI 26–39 kg/m^2^ (2.8%, 1.6%, 1.4%) in the obese groups, and then increase again in morbid obese patients, 1.6% (Figure 1).

**Figure 2:**
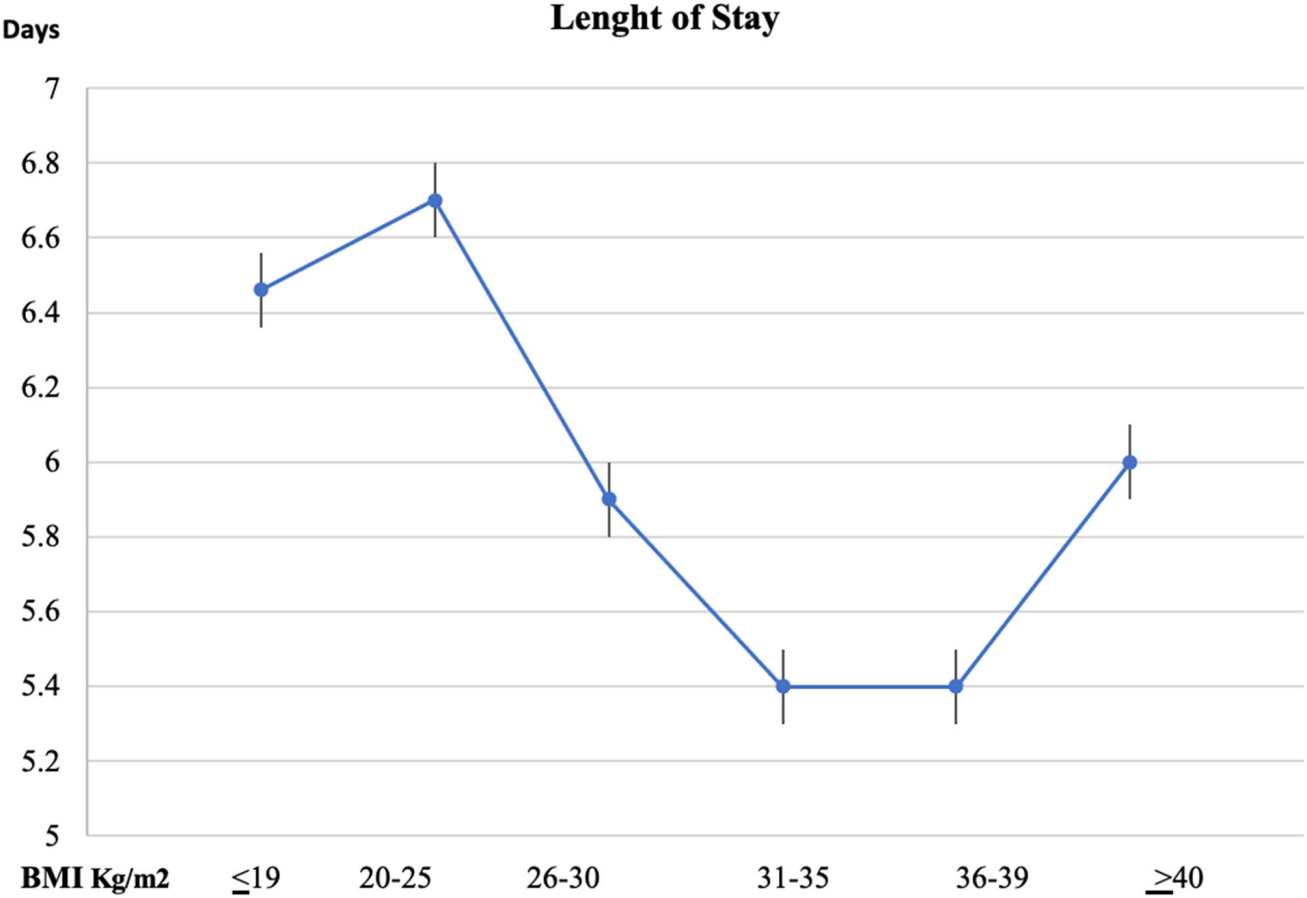
Length of Stay *via* BMI groups.

Length of stay, like the mortality, was found to be greater in BMI < 19 (6.46 + 0.15), BMI 20–25 (6.7 + 0.15), and BMI > 40 (6.0 + 0.05) (Figure 2).

### Predictors of In-hospital Mortality

In the univariate analysis, we found a reverse J-shape association between survival with increasing BMI with lower mortality rate in obese patients with BMI > 25 kg/m^2^.

Other baseline characteristics, such as older age, increasing Deyo-CCI score, white race, atrial fibrillation/flutter, peripheral vascular disease, and chronic renal failure, all increased the probability of in-hospital mortality (*p* < 0.001) (Table 2). In contrast, diabetes mellitus and hypertension were two protective comorbidities against mortality in patients with AHF (*p* < 0.001).

**Table 2:** Univariate analysis for predictors of in-hospital mortality, 2015–2016.

**Predictor**	**Odds ratio (95% CI)**	***P*-value**
**BMI kg/m**^**2**^ **group**		<0.001
≤ 19	0.99 (0.89,1.10)	0.786
20–25	1.00 (reference)	N/A
26–30	0.49 (0.43,0.55)	<0.001
31–35	0.28 (0.25,0.31)	<0.001
36–39	0.24 (0.21,0.27)	<0.001
≥40	0.28 (0.26,0.31)	<0.001
**Age group, years**		<0.001
18–44	1.00 (reference)	N/A
45–59	1.54 (1.28,1.86)	<0.001
60–74	2.08 (1.74,2.48)	<0.001
≥75	4.43 (3.72,5.27)	<0.001
**Deyo-CCI**		<0.001
0	N/A	N/A
1	1.00 (reference)	N/A
2 or higher	1.39 (1.19,1.62)	<0.001
**Gender**		0.169
Male	1.00 (reference)	N/A
Female	1.04 (0.98,1.10)	0.169
**Race**		<0.001
Non-white	1.00 (reference)	N/A
White	1.74 (1.63,1.87)	<0.001
***Comorbidities***		
**Atrial fibrillation/Flutter**		<0.001
No	1.00 (reference)	N/A
Yes	1.90 (1.79,2.01)	<0.001
**Chronic pulmonary disease**		0.001
No	1.00 (reference)	N/A
Yes	0.91 (0.86,0.96)	0.001
**Diabetes mellitus**		<0.001
No	1.00 (reference)	N/A
Yes	0.73 (0.69,0.77)	<0.001
**Hypertension**		<0.001
No	1.00 (reference)	N/A
Yes	0.53 (0.50,0.57)	<0.001
**Peripheral vascular disease**		<0.001
No	1.00 (reference)	N/A
Yes	1.30 (1.22,1.39)	<0.001
**Prior MI**		0.670
No	1.00 (reference)	N/A
Yes	0.98 (0.90,1.07)	0.670
**Chronic renal failure**		<0.001
No	1.00 (reference)	N/A
Yes	1.53 (1.44,1.62)	<0.001

*BMI, Body Mass Index; Deyo-CCI, Deyo-Charlson Comorbidity Index; MI, Myocardial Infraction*.

The reverse J-shaped correlation between BMI and mortality outcomes was again identified in the multivariable analyses (Table 3). A similar trend to the univariate model was also identified in the multivariate model of baseline characteristics. Older age >75 years, Deyo-CCI >2, white race, atrial fibrillation/flutter, peripheral vascular disease, and chronic renal failure all remained strong predictors of mortality after adjusting for potential confounders (Table 3). Furthermore, diabetes mellitus and hypertension remained protective against mortality (*p* < 0.001).

**Table 3:** Multivariable analysis for predictors of in-hospital mortality, 2015–2016.

**Predictor**	**Odds ratio (95% CI)**	***P*-value**
**BMI kg/m**^**2**^ **group**		<0.001
≤ 19	0.96 (0.86,1.07)	0.487
20–25	1.00 (reference)	N/A
26–30	0.55 (0.49,0.62)	<0.001
31–35	0.33 (0.29,0.37)	<0.001
36–39	0.29 (0.25,0.33)	<0.001
≥40	0.41 (0.37,0.45)	<0.001
**Age group, years**		<0.001
18–44	1.00 (reference)	N/A
45–59	1.52 (1.25,1.84)	<0.001
60–74	1.81 (1.50,2.18)	<0.001
>75	3.09 (2.56,3.73)	<0.001
**Deyo-CCI**		<0.001
1	1.00 (reference)	N/A
2 or higher	1.67 (1.41–1.97)	<0.001
**Gender**		0.004
Male	1.00 (reference)	N/A
Female	0.91 (0.86,0.97)	0.004
**Race**		<0.001
Non-white	1.00 (reference)	N/A
White	1.47 (1.36,1.59)	<0.001
***Comorbidities***		
**Atrial fibrillation/Flutter**		<0.001
No	1.00 (reference)	N/A
Yes	1.48 (1.39,1.58)	<0.001
**Chronic pulmonary disease**		0.046
No	1.00 (reference)	N/A
Yes	0.94 (0.88,1.00)	0.046
**Diabetes mellitus**		<0.001
No	1.00 (reference)	N/A
Yes	0.86 (0.81,0.92)	<0.001
**Hypertension**		<0.001
No	1.00 (reference)	N/A
Yes	0.59 (0.55,0.63)	<0.001
**Peripheral vascular disease**		<0.001
No	1.00 (reference)	N/A
Yes	1.16 (1.08,1.24)	<0.001
**Prior MI**		0.050
No	1.00 (reference)	N/A
Yes	0.92 (0.84,1.00)	0.050
**Chronic renal failure**		<0.001
No	1.00 (reference)	N/A
Yes	1.46 (1.37,1.55)	<0.001

*BMI, Body Mass Index; Deyo-CCI, Deyo-Charlson Comorbidity Index; MI, Myocardial Infraction*.

### Predictors of Length of Stay (LOS)

The multivariable regression model for LOS is represented in Table 4. A similar reverse J-shape slope to that of mortality was observed for LOS, with the highest value for normal weight BMI 20–25 kg/m^2^ [OR 5.51 (95% CI 5.24–5.78)] and lowest values for obese patients [OR 4.20 (95% CI 4.01–4.40), OR 4.24 (95% CI 4.04–4.44)] for BMI 31–35 kg/m^2^ and 36–39 kg/m^2^ obese groups, respectively, *p* < 0.001, (Table 4).

**Table 4:** Multivariable analysis for predictors of length of stay (LOS), 2015–2016.

**Predictor**	**Odds ratio (95% CI)**	***P*-value**
**Age group, years**		<0.001
18–44	4.47 (4.22,4.72)	N/A
45–59	4.81 (4.62,4.99)	0.005
60–74	5.03 (4.86,5.20)	<0.001
>75	4.84 (4.68,5.00)	0.003
**BMI kg/m**^**2**^ **group**		<0.001
<19	5.09 (4.83,5.35)	0.012
20–25	5.51 (5.24,5.78)	N/A
26–30	4.78 (4.55,5.02)	<0.001
31–35	4.20 (4.01,4.40)	<0.001
36–39	4.24 (4.04,4.44)	<0.001
>40	4.89 (4.74,5.04)	<0.001
**Gender**		0.007
Female	4.86 (4.70,5.03)	0.007
Male	4.71 (4.55,4.87)	N/A
**Race**		0.872
Non-white	4.78 (4.61,4.95)	N/A
White	4.79 (4.64,4.95)	0.872
**Deyo-CCI**		<0.001
1	4.22 (3.96,4.48)	N/A
2 or higher	5.35 (5.24,5.46)	<0.001
***Comorbidities***		
**Atrial fibrillation/Flutter**		<0.001
No	4.50 (4.34,4.65)	N/A
Yes	5.35 (5.18,5.52)	<0.001
**Chronic pulmonary disease**		0.563
No	4.78 (4.63,4.93)	N/A
Yes	4.81 (4.64,4.99)	0.563
**Diabetes mellitus**		<0.001
No	4.84 (4.68,4.99)	N/A
Yes	4.51 (4.33,4.69)	<0.001
**Hypertension**		<0.001
No	5.41 (5.24,5.57)	N/A
Yes	4.36 (4.20,4.51)	<0.001
**Prior MI**		<0.001
No	4.83 (4.68,4.98)	N/A
Yes	4.25 (4.04,4.46)	<0.001
**Peripheral vascular disorders**		0.492
No	4.78 (4.63,4.93)	N/A
Yes	4.83 (4.63,5.02)	0.492
**Renal failure**		<0.001
No	4.61 (4.46,4.76)	N/A
Yes	5.51 (5.34,5.69)	<0.001

*BMI, Body Mass Index; Deyo-CCI, Deyo-Charlson Comorbidity Index; MI, Myocardial Infraction*.

## Discussion

In this retrospective study benefiting from the NIS, the largest all-payer in-patient database in the US, we distinguished 219,950 patients to study the correlation between BMI and in-hospital mortality and LOS outcomes following AHF hospitalization. To our knowledge, this is a distinct main study examining the relationship between BMI and in-hospital mortality in patients with AHF.

This nationally analyzed dataset indicated a reverse J-shape correlation between BMI levels and in-hospital mortality during hospitalization of patients with AHF across the US during the years 2015–2016.

In patients that were hospitalized with AHF, elevated BMI was found to be an independent predictor of lower in-hospital mortality and shorter length of stay during the study period.

Lower mortality rates were detected in the BMI 31–35 kg/m^2^ and BMI 36–39 kg/m^2^ obese groups after correcting for confounding variables.

Body mass index is proven to be an independent risk factor for various cardiovascular conditions, such as atrial and ventricular arrhythmia, sudden cardiac death, stroke, acute coronary syndrome, and HF (23–25). Overweight and obesity were found to be risk factors for left ventricular remodeling and overt HF (23). Obesity has been consistently associated with left ventricular hypertrophy and dilatation, which are known precursors of HF (26–28).

Prior studies in patients with AHF examined the effect of different BMI subgroups on all-cause mortality and presented a U-shape prototype, with lowest mortality in the obese and the underweight patient populations (7–17). These studies suggested an obesity paradox in which BMI does not distinguish between metabolically healthy and metabolically unhealthy obesity (3). The obesity impact on morbidity and premature mortality can be underestimated and therefore may lead to incorrect clinical courses (3). Studies suggest that the obesity paradox is present in HF with preserved ejection fraction (HFpEF) and HF with reduced ejection fraction (HFrEF) (29). A potential explanation is that BMI represents lean body mass rather than accurate body fat or fluid retention in patients with HF (2). In a recent study of HF, obesity was associated with a reduced risk of death, but this protective effect disappeared after adjusting for VO_2_ max and b-type natriuretic peptide (BNP) levels (30).

In contrast with other studies that showed lower mortality rates in underweight patients (7–17), in line with our findings, Seko et al. also revealed that very low BMI was associated with a higher risk of mortality in patients with AHF (15). In their study, greater mortality risk was observed in underweight and severely underweight patients with AHF compared to the normal weight BMI, which remained significant even after adjusting confounders, while the lower mortality risk in the overweight and in the obese groups was no longer significant after these adjustment (15). The results of this study demonstrate the concept that the deleterious effect of cachexia, rather than the favorable influence of obesity [in the form of nutritional and caloric reserve based on several studies (31–33)], are likely the main reason for the inverse correlation between BMI and HF outcome (4). In severe HF, tissue hypoperfusion and cardiac cachexia might contribute to the adverse outcomes (34). We cannot explain the increase in mortality and length of stay in the extremely obese, as they had similar comorbidities. A focus on this BMI sub-group should be the goal of future studies to better understand the mechanisms behind this phenomenon. Also, an additional investigation using body fat mass might add insight to these findings.

A few mechanisms may explain the obesity paradox in acute HF. It appears that higher lean body mass may be protective by role of myocytes on vasculature and by favorable cytokines (2). Fat tissue has been shown to produce tumor necrosis factor-α receptors which may be protective (2). High circulating lipoprotein levels in obese subjects may bind and detoxify lipopolysaccharides that may play a role in the release of inflammatory cytokines (35, 36). Experimental studies suggested that leptin produced by fat tissue may have a protective effect in HF (37). In obesity, lower levels of adiponectin were detected, associating it with lower mortality in HF (38). Also, there might be a higher use of guidelines-guided therapy in patients with higher BMI, according to one study (9). Taken together, fat distribution, lean mass, and cardio fitness could play essential roles in determining the observed differences in the HF population (3).

This study has several limitations. The accessible NIS database is retrospective and, as such, contains discharge patient records which are susceptible to coding errors. The NIS dataset lacks comprehensive information regarding medication, blood testing, and other important markers, such as NT-proBNP, that are associated with adverse cardiovascular events which are markedly important confounding variables. We therefore cannot rule out residual confounding of the associations we observed. Furthermore, we could only capture events that occurred in the same index hospitalization. Our model did not check collinearity between the dependent variable. As such, we cannot exclude the multicollinearity of these variables. These limitations are compensated by the real world, nationwide disposition of the data, and modification of reporting bias introduced by selective publication of results from specialized centers.

In conclusion, our study results improve the available literature on the protective association of obese BMI subgroups with mortality following AHF hospitalization. We showed a reverse J-shaped relationship between BMI and mortality. Higher BMI was independently associated with protecting against mortality and decreasing LOS, while the mild and moderately obese patient subgroups (BMI 31–39) exhibited the lowest in-hospital mortality in this study. Accordingly, BMI should be addressed carefully and considered by differences in clinical profile and treatment and not solely by an effect of body composition as part of the risk assessment for in-hospital mortality in patients who are hospitalized for AHF.

## Data Availability Statement

The Publicly available datasets were analyzed in this study. This data can be found here: Agency for Healthcare research and quality, Nis database, link : hcup-us.ahrg.gov.

## Ethics Statement

Ethical review and approval was not required for the study on human participants in accordance with the local legislation and institutional requirements. Written informed consent for participation was not required for this study in accordance with the national legislation and the institutional requirements.

## Author Contributions

GE-G and GR: conceived the idea and design of the study and drafted the paper. MY: drafted the paper. SC: data analysis and interpretation and provided revisions to the manuscript. HW: data interpretation and provided major revisions to the manuscript. DP, ER, IG, and RA: major provided revisions to the manuscript. OA: conceived the idea and design of the study, principal investigator, and provided revisions to the manuscript. All authors had major contribution.

## Conflict of Interest

The authors declare that the research was conducted in the absence of any commercial or financial relationships that could be construed as a potential conflict of interest.

## Publisher's Note

All claims expressed in this article are solely those of the authors and do not necessarily represent those of their affiliated organizations, or those of the publisher, the editors and the reviewers. Any product that may be evaluated in this article, or claim that may be made by its manufacturer, is not guaranteed or endorsed by the publisher.
